# ACRATA: a novel electron transfer domain associated to apoptosis and cancer

**DOI:** 10.1186/1471-2407-4-98

**Published:** 2004-12-29

**Authors:** Luis Sanchez-Pulido, Ana M Rojas, Alfonso Valencia, Carlos Martinez-A, Miguel A Andrade

**Affiliations:** 1 Protein Design Group, Centro Nacional de Biotecnología (CNB-CSIC), Cantoblanco, Madrid, E-28049, Spain; 2 Departamento de Inmunologia y Oncologia DIO, Centro Nacional de Biotecnología (CNB-CSIC), Cantoblanco, Madrid, E-28049, Spain; 3 Ontario Genomics Innovation Centre, Ottawa Health Research Institute, 501 Smyth Road, Box 411, Ottawa, ON K1H 8L6, Canada; 4 Department of Cellular and Molecular Medicine, Faculty of Medicine, University of Ottawa, Ottawa, Canada

## Abstract

**Background:**

Recently, several members of a vertebrate protein family containing a six trans-membrane (6TM) domain and involved in apoptosis and cancer (e.g. STEAP, STAMP1, TSAP6), have been identified in Golgi and cytoplasmic membranes. The exact function of these proteins remains unknown.

**Methods:**

We related this 6TM domain to distant protein families using intermediate sequences and methods of iterative profile sequence similarity search.

**Results:**

Here we show for the first time that this 6TM domain is homolog to the 6TM heme binding domain of both the NADPH oxidase (Nox) family and the YedZ family of bacterial oxidoreductases.

**Conclusions:**

This finding gives novel insights about the existence of a previously undetected electron transfer system involved in apoptosis and cancer, and suggests further steps in the experimental characterization of these evolutionarily related families.

## Background

A family of vertebrate proteins containing a six transmembrane domain (6TM) has been recently implicated in apoptosis and cancer. Up to date, this family contains four vertebrate members: STEAP, STAMP1/STEAP2, TIARP and TSAP6/pHyde. STEAP (six-transmembrane epithelial antigen of the prostate) was the first described member of this family and identified as a prostate-specific cell-surface antigen overexpressed in cancer, located at the cell-cell junction of the secretory epithelium of prostate, and found as well in both colon and bladder cancer cell lines [1,2]. STAMP1 (six transmembrane protein of prostate 1), also known as STEAP2, also overexpressed in prostate cancer, has been located in the trans-Golgi network and shuttles to plasma membranes, which suggest a role in the secretory/endocytic pathways [3,4]. TIARP (Tumor necrosis factor-alpha-induced adipose-related protein) is a cell surface protein induced by TNF-α and IL-6, probably implicated in resistance to insulin [5,6]. TSAP6 (tumor suppressor activated pathway-6), also known as pHyde [7-10], is a p53 inducible protein which regulates apoptosis and the cell cycle via direct interaction with Nix (a pro-apoptotic Bcl-2 related protein) and Myt1 kinase (a negative regulator of the G2/M transition) [9]. TSAP6 has been shown to be interacting with TCTP (Translationally controlled tumor protein) and could be implicated in its secretion [10].

**Figure 1:**
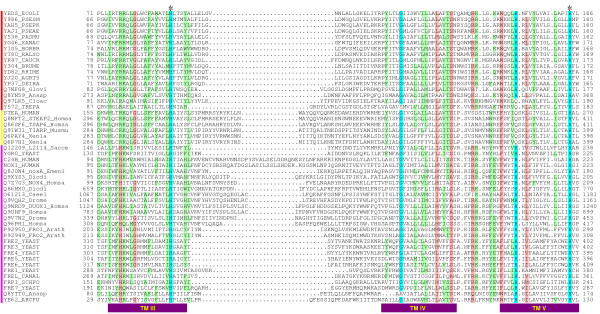
**Representative multiple alignment of the ACRATA domain. **It is viewed with the Belvu program [33]. The colouring scheme indicates average BLOSUM62 score (correlated to amino acid conservation) in each alignment column: cyan (greater than 3), light red (between 3 and 1.5) and light green (between 1.5 and 0.5). The limits of the domains are indicated by the residue positions on each side. The TMHMM helix transmembrane [29, 30] consensus prediction is shown below the alignment. The asterisks above the alignment mark the conserved histidine residues mentioned in the text. Different groups of the ACRATA sequences are shown by coloured lines to the left of the alignment: red, YedZ family; yellow, STEAP family; violet, Nox family. The sequences are named with their swissprot or sptrembl identifiers, and also, if necessary, with their gene name. Species abbreviations: Homsa, *Homo sapiens*; Glovi, *Gloeobacter violaceus*; Anasp, *Anabaena sp*.; Cloac, *Clostridium acetobutylicum*; Musmu, *Mus musculus*; Xenla, *Xenopus laevis*; Sacce, *Saccharomyces cerevisiae*; Emeni, *Emericella nidulans*; Dicdi, *Dictyostelium discoideum*; Caeel, *Caenorhabditis elegans*; Drome, *Drosophila melanogaster*; Arath, *Arabidopsis thaliana*. Complementary information is accessible at: .

**Figure 2:**
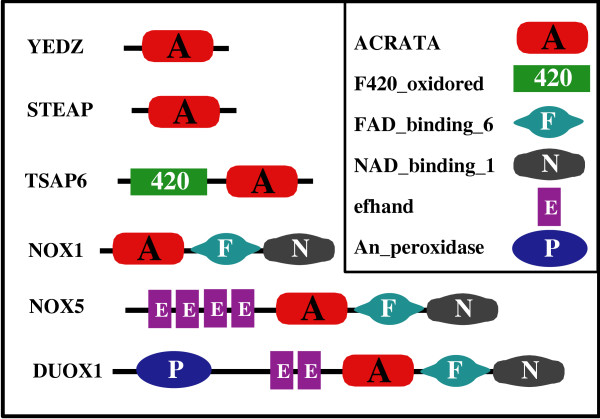
**Schematic representation of the domain architecture and common features in representative proteins containing the ACRATA domain. **The representative sequences selected correspond to: YEDZ, SW:YEDZ_ECOLI; STEAP, SW:STEA_HUMAN; TSAP6, SP:Q80ZF3; NOX1, SW:NOX1_HUMAN; NOX5, SP:Q96PH2; DUOX1, SP:Q9NRD9. NOX1, NOX5, and DUOX1, belong to the Nox family. The proteins are drawn approximately to scale. The domains are named and located according to the Pfam and SMART protein domain databases [34-37]. Abbreviations: SP, SPTREMBL; SW, SWISSPROT.

In this work we present evidence of remote homology of this family to other two families: the mainly eukaryotic Nox and the bacterial YedZ (Fig. 1), both involved in redox functions [11-17]. The Nox family is involved in the production of reactive oxygen species (ROS) [11,12]. The first member of the family (gp91phox) was discovered in phagocytes and contains an N-terminal transmembrane heme binding domain and two C-terminal domains with binding sites for both flavin adenine dinucleotide (FAD) and NADPH (Fig. 2) [11-13]. Originally, ROS were thought to be used just as a mechanism of host defence. The discovery of gp91phox homologues in several other tissues has suggested their implication in many other functions, such us signal transduction, cancer, mitogenic signalling, cellular growth, angiogenesis, and modification of extracellular matrix proteins [11-14]. Regarding the bacterial YedZ family, the only experimentally characterized member so far is the *Escherichia coli *YedZ protein, which binds a single heme and is involved in electron transfer to the molybdopterin cofactor in YedY, its operon neighbour gene [16-18]. The exact function of the operon YedZ/YedY remains unknown.

## Methods

To do the sequence analysis of the new domain we took advantage of the possibility of connecting distant protein families via intermediate sequences [19] and methods of iterative profile sequence similarity search: HMMer [20,21] and PSI-BLAST [22] over the Uniprot 90% non redundant sequence database [23]. We used NAIL to view and analyse the HMMer results [24]. The alignment of transmembrane regions using standard substitution matrices might be inaccurate because of the different roles played by amino acids in globular proteins and in transmembrane media [25]. In the case of the ACRATA domain, the regions to be aligned mostly consist of amino acids located in transmembrane regions (Fig. 1). For this reason, we used a method based on a hidden Markov model (HMMer hmmalign), which does not rely on a general substitution matrix [20,21], using as a guide both the transmembrane predictions from TMHMM [26,27] and the results of multiple sequence alignment using T-Coffee [28,29]. The genomic neighborhood of the bacterial sequences (YedZ family) was analyzed to find potentially related genes in operons using STRING [17,18].

## Results

**Figure 3:**
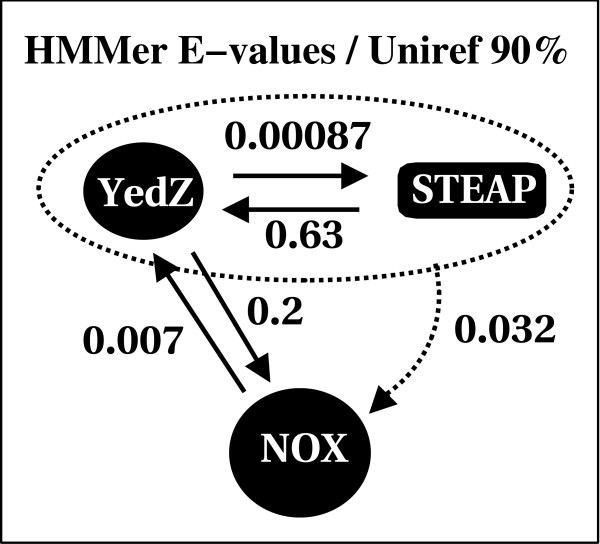
**HMMer E-Values between the ACRATA domain containing families. **The numbers correspond to HMMer E-values from global profile search results [20, 21] that connect independently each family with the others. The arrows indicate the profile search direction, for example: the YedZ family profile search finds sequences of the STEAP family with a 0.00087 E-value and the global profile of the YedZ/STEAP families (dotted line) detected the Nox family with an E-value of 0.032.

The global hidden Markov profile [20,21] generated for STEAP and related vertebrate proteins (STEAP family, henceforth) localized the first bacterial sequence (SpTrembl Q7VKI9 from *Haemophilus ducreyi*) with an E-value of 0.63. This protein belongs to the large YedZ family of bacterial oxidoreductases. The corresponding YedZ global profile detected the STEAP family (most similar member: SpTrembl Q8IUE7, human STAMP1) with an E-value of 0.00087. The global profile of STEAP and YedZ detected the Nox family with an E-value of 0.032 and the corresponding Nox global profile localized the YedZ family with an E-value of 0.007 (Fig. 3). Only the regions from transmembranes 3 to 5 were considered to build the profiles because the transmembranes 1, 2 and 6 are highly variable among families. We have named this 6TM domain the ACRATA domain after **A**poptosis, **C**ancer and **R**edox **A**ssociated **T**ransmembr**A**ne domain.

To investigate the consistency of our results we performed iterative database searches using the PSI-BLAST program [22]. We used as query the most conserved region of the ACRATA domain in *E. coli *YedZ protein (residues 71–166). These searches detected all of the ACRATA domain-containing families after 15 iterations (using a cut-off of E = 0.005 for the inclusion of retrieved sequences in the profile). None of these profile searches retrieved new unrelated sequences, and reciprocal searches produced convergent results. Therefore, we have concluded that ACRATA is a previously undetected, conserved domain that is commonly found in members of the STEAP, YedZ and Nox protein families. The similarity between these proteins was suspected before [30]. However, no alignment, domain definition or substantial statistical evidence was provided to demonstrate the evolutionary relationships of these proteins.

The complete conservation of two histidines in all ACRATA domain containing proteins (Fig. 1) indicates that the STEAP protein family could bind at least an heme group, as was previously experimentally characterized for Nox and YedZ families [13,16], or for other analogous proteins such as cytochromes [31].

## Discussion

**Figure 4:**
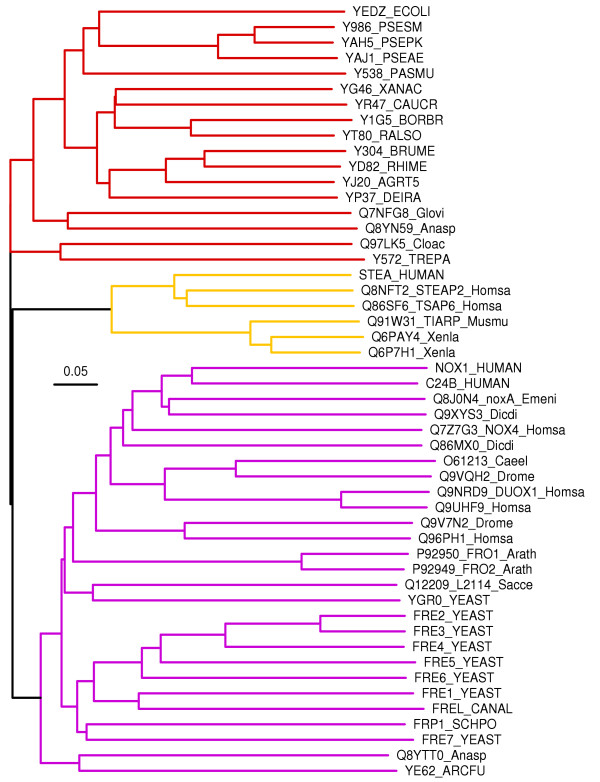
**Phylogenetic tree of selected ACRATA domain containing proteins. **The scale bar represents the number of inferred substitutions per 100 sites (amino acid residues). The tree branches for YedZ proteins are in red, STEAP branches are in yellow, and Nox branches are in violet. Organism name abbreviations are as in Fig. 1. We generated trees using a Bayesian, Neighbor Joining, and Minimum Evolution methods, and found that all three gave the same topology suggesting that the overall structure of the tree is correct. For illustrative purposes only the Neighbor Joining tree is shown [38-40].

Experimental evidence show that Nox and YedZ families share heme binding capabilities and also involvement in electron transfer chains [11-13,16]. For the STEAP family, the electron transfer capability is consistent with the presence of an N-terminal cytoplasmic NADP oxidoreductase coenzyme F420 dependent domain, being the only exception the STEAP protein itself (Fig. 2). Therefore we conclude that ACRATA domain is a heme binding 6TM domain that originated before the onset of eukaryotes (ancestral, YedZ), transmitted by vertical descent in a conventional manner (Nox family), and further expanded in vertebrates (STEAP family) (Fig. 4).

Although the mechanism of action of the ACRATA domain could be the same in all the proteins containing it, its variable cellular role is made conspicuous by the different effects produced by the modifications in the expression of the corresponding genes. For example, the knock-out of the whole YedYZ operon seems not to affect *E. coli *in a number of conditions tested (Brokx, S.J. and Weiner, J.H. personal communication). Very differently, changes in the expression patterns of human genes containing the ACRATA domain could be related with apoptosis or cancer [1-14]. The well known functional flexibility of oxidoreductases is exemplified in bacterial proteins such as cupredoxins and cytochromes, normally involved in electron transfer during respiration but that can enter in eukaryotic cells to induce apoptosis or inhibition of cell growth [32], or in the Nox family, with functions as different as host defence in phagocytes or extracellular matrix modification [11,12].

## Conclusions

We have described for the first time a 6TM domain present in three protein families (STEAP, YedZ and Nox): the ACRATA domain. The common functions of the proteins of those families suggest that this domain is involved in electron transfer, mediated by its heme binding capability. We hypothesize that STEAP, STAMP1, TSAP6, and TIARP have this function, and that they form part of electron transfer systems involved in cellular regulation, apoptosis, and cancer. Additional experimental approaches using different members of the ACRATA domain-containing families are required to confirm these hypotheses.

## Abbreviations

STEAP, six-transmembrane epithelial antigen of the prostate;

STAMP1, six transmembrane protein of prostate 1;

TSAP6, tumor suppressor activated pathway-6;

TIARP, Tumor necrosis factor-alpha-induced adipose-related protein;

TCTP, Translationally controlled tumor protein;

Nox, NADPH oxidase;

ROS, reactive oxygen species;

TM, Trans-membrane;

FAD, flavin adenine dinucleotide;

HMM, Hidden Markov Models;

## Competing interests

The author(s) declare that they have no competing interests.

## Authors' contributions

LSP, AMR, and MAA carried out the sequence analysis of the domain.

LSP and MAA provided with the initial input of the research.

LSP, AMR, AV, CMA, and MAA authored the manuscript.

## Pre-publication history

The pre-publication history for this paper can be accessed here:



## References

[B1] Hubert RS, Vivanco I, Chen E, Rastegar S, Leong K, Mitchell SC, Madraswala R, Zhou Y, Kuo J, Raitano AB, Jakobovits A, Saffran DC, Afar DE (1999). STEAP: a prostate-specific cell-surface antigen highly expressed in human prostate tumors. Proc Natl Acad Sci U S A.

[B2] Yang D, Holt GE, Velders MP, Kwon ED, Kast WM (2001). Murine six-transmembrane epithelial antigen of the prostate, prostate stem cell antigen, and prostate-specific membrane antigen: prostate-specific cell-surface antigens highly expressed in prostate cancer of transgenic adenocarcinoma mouse prostate mice. Cancer Res.

[B3] Korkmaz KS, Elbi C, Korkmaz CG, Loda M, Hager GL, Saatcioglu F (2002). Molecular cloning and characterization of STAMP1, a highly prostate-specific six transmembrane protein that is overexpressed in prostate cancer. J Biol Chem.

[B4] Porkka KP, Helenius MA, Visakorpi T (2002). Cloning and characterization of a novel six-transmembrane protein STEAP2, expressed in normal and malignant prostate. Lab Invest.

[B5] Moldes M, Lasnier F, Gauthereau X, Klein C, Pairault J, Feve B, Chambaut-Guerin AM (2001). Tumor necrosis factor-alpha-induced adipose-related protein (TIARP), a cell-surface protein that is highly induced by tumor necrosis factor-alpha and adipose conversion. J Biol Chem.

[B6] Fasshauer M, Kralisch S, Klier M, Lossner U, Bluher M, Chambaut-Guerin AM, Klein J, Paschke R (2004). Interleukin-6 is a positive regulator of tumor necrosis factor alpha-induced adipose-related protein in 3T3-L1 adipocytes. FEBS Lett.

[B7] Zhang X, Steiner MS, Rinaldy A, Lu Y (2001). Apoptosis induction in prostate cancer cells by a novel gene product, pHyde, involves caspase-3. Oncogene.

[B8] Porkka KP, Nupponen NN, Tammela TL, Vessella RL, Visakorpi T (2003). Human pHyde is not a classical tumor suppressor gene in prostate cancer. Int J Cancer.

[B9] Passer BJ, Nancy-Portebois V, Amzallag N, Prieur S, Cans C, Roborel de Climens A, Fiucci G, Bouvard V, Tuynder M, Susini L, Morchoisne S, Crible V, Lespagnol A, Dausset J, Oren M, Amson R, Telerman A (2003). The p53-inducible TSAP6 gene product regulates apoptosis and the cell cycle and interacts with Nix and the Myt1 kinase. Proc Natl Acad Sci U S A.

[B10] Amzallag N, Passer BJ, Allanic D, Segura E, Thery C, Goud B, Amson R, Telerman A (2004). TSAP6 facilitates the secretion of TCTP/HRF via a non-classical pathway. J Biol Chem.

[B11] Lambeth JD (2004). NOX enzymes and the biology of reactive oxygen. Nat Rev Immunol.

[B12] Geiszt M, Leto TL (2004). The Nox family of NAD(P)H oxidases: Host defense and beyond. J Biol Chem.

[B13] Yu L, Quinn MT, Cross AR, Dinauer MC (1998). Gp91(phox) is the heme binding subunit of the superoxide-generating NADPH oxidase. Proc Natl Acad Sci U S A.

[B14] Mitsushita J, Lambeth JD, Kamata T (2004). The superoxide-generating oxidase Nox1 is functionally required for Ras oncogene transformation. Cancer Res.

[B15] Drew D, Sjostrand D, Nilsson J, Urbig T, Chin CN, de Gier JW, von Heijne G (2002). Rapid topology mapping of Escherichia coli inner-membrane proteins by prediction and PhoA/GFP fusion analysis. Proc Natl Acad Sci U S A.

[B16] Loschi L, Brokx SJ, Hills TL, Zhang G, Bertero MG, Lovering AL, Weiner JH, Strynadka NC (2004). Structural and biochemical identification of a novel bacterial oxidoreductase. J Biol Chem.

[B17] von Mering C, Huynen M, Jaeggi D, Schmidt S, Bork P, Snel B (2003). STRING: a database of predicted functional associations between proteins. Nucleic Acids Res.

[B18] STRING. http://string.embl.de/.

[B19] Park J, Teichmann SA, Hubbard T, Chothia C (1997). Intermediate sequences increase the detection of homology between sequences. J Mol Biol.

[B20] Eddy SR (1998). Profile hidden Markov models. Bioinformatics.

[B21] HMMer. http://hmmer.wustl.edu/.

[B22] Altschul SF, Madden TL, Schaffer AA, Zhang J, Zhang Z, Miller W, Lipman DJ (1997). Gapped BLAST and PSI-BLAST: a new generation of protein database search programs. Nucleic Acids Res.

[B23] Apweiler R, Bairoch A, Wu CH (2004). Protein sequence databases. Curr Opin Chem Biol.

[B24] Sanchez-Pulido L, Yuan YP, Andrade MA, Bork P (2000). NAIL-Network Analysis Interface for Linking HMMER results. Bioinformatics.

[B25] Jones DT, Taylor WR, Thornton JM (1994). A mutation data matrix for transmembrane proteins. FEBS Lett.

[B26] Krogh A, Larsson B, von Heijne G, Sonnhammer EL (2001). Predicting transmembrane protein topology with a hidden Markov model: application to complete genomes. J Mol Biol.

[B27] TMHMM. http://www.cbs.dtu.dk/services/TMHMM.

[B28] Notredame C, Higgins DG, Heringa J (2000). T-Coffee: A novel method for fast and accurate multiple sequence alignment. J Mol Biol.

[B29] T-COFFEE. http://www.ch.embnet.org/software/TCoffee.html.

[B30] TCDB. http://tcdb.ucsd.edu.

[B31] Walker FA (2004). Models of the Bis-Histidine-Ligated Electron-Transferring Cytochromes. Comparative Geometric and Electronic Structure of Low-Spin Ferro- and Ferrihemes. Chem Rev.

[B32] Yamada T, Hiraoka Y, Das Gupta TK, Chakrabarty AM (2004). Regulation of mammalian cell growth and death by bacterial redox proteins: relevance to ecology and cancer therapy. Cell Cycle.

[B33] BELVU. http://www.sanger.ac.uk/Software/Pfam/help/belvu_setup.shtml.

[B34] Bateman A, Coin L, Durbin R, Finn RD, Hollich V, Griffiths-Jones S, Khanna A, Marshall M, Moxon S, Sonnhammer EL, Studholme DJ, Yeats C, Eddy SR (2004). The Pfam protein families database. Nucleic Acids Res.

[B35] PFAM. http://www.sanger.ac.uk/Software/Pfam/.

[B36] Letunic I, Copley RR, Schmidt S, Ciccarelli FD, Doerks T, Schultz J, Ponting CP, Bork P (2004). SMART 4.0: towards genomic data integration. Nucleic Acids Res.

[B37] SMART. http://smart.embl.de/.

[B38] Feng DF, Doolittle RF (1996). Progressive alignment of amino acid sequences and construction of phylogenetic trees from them. Methods Enzymol.

[B39] Kumar S, Tamura K, Jakobsen IB, Nei M (2001). MEGA2: molecular evolutionary genetics analysis software. Bioinformatics.

[B40] Ronquist F, Huelsenbeck JP (2003). MrBayes 3: Bayesian phylogenetic inference under mixed models. Bioinformatics.

